# HLA-DMB correlates with antitumor immunity and an improved prognosis in endometrial carcinoma tumors

**DOI:** 10.3389/fonc.2024.1525601

**Published:** 2025-01-23

**Authors:** Xiaoyu Xi, Xiaona Zhang, Jianxin Tang, Xiumei Fan, Jiexian Du

**Affiliations:** ^1^ Department of Gynecology, The Second Hospital of Hebei Medical University, Shijiazhuang, Hebei, China; ^2^ Department of Gynecology, Hengshui Fifth People’s Hospital, Hengshui, Hebei, China; ^3^ Department of Obstetrics and Gynecology, Xingtai Third Hospital, Xingtai, Hebei, China

**Keywords:** endometrial carcinoma, prognosis, immunohistochemistry, anti-tumor immunity, qPCR

## Abstract

**Introduction:**

Endometrial Carcinoma (UCEC) is a prevalent malignant tumor within the female reproductive system. HLA-DMB, the beta chain of the non-classical MHC class II protein HLA-DM, has been implicated in the progression of various cancers. However, its role in the development of endometrial carcinoma remains unclear. Therefore, we conducted a preliminary exploration of the prognostic value and potential mechanisms of HLA-DMB in uterine corpus endometrial carcinoma (UCEC).

**Methods:**

The differential expression of HLA-DMB was analyzed in 554 tumor samples and 35 normal samples obtained from the TCGA database. The differential expression of HLA-DMB across various cancers, along with immune infiltration analysis, was conducted using the TIMER2.0 database. Additionally, the expression of HLA-DMB in endometrial carcinoma was examined in the GEPIA2 database, along with its relationship to prognosis. Furthermore, TISIDB was utilized to predict the relationships between HLA-DMB and various immune enhancement factors as well as immunosuppressive factors. Gene Ontology (GO) analysis and Gene Set Enrichment Analysis (GSEA) were employed to explore the signaling pathways associated with HLA-DMB in endometrial cancer. Univariate COX regression analysis was performed to identify prognostic factors for endometrial carcinoma (EC), and a multivariate COX proportional hazards regression model was used to confirm that HLA-DMB can serve as an independent prognostic factor for EC. The protein interaction network of HLA-DMB was constructed using the STRING database, and the chemical drugs related to HLA-DMB were predicted through the CTD database. Finally, the expression of HLA-DMB was validated by qPCR and immunohistochemistry.

**Results:**

The expression of HLA-DMB at both mRNA and protein levels is significantly higher in UCEC tissues compared to normal tissues. Prognostic analyses indicate that increased expression of HLA-DMB correlates with improved patient prognosis, suggesting its potential as an independent prognostic factor for UCEC. Furthermore, in endometrial cancer, elevated levels of HLA-DMB are associated with higher immune infiltration scores and are closely related to various immune-enhancing factors. Mechanistically, HLA-DMB primarily participates in CD22-mediated regulation of B cell receptors (BCR), leading to BCR antigen activation and the production of second messengers. In our drug analysis, we identified several chemical agents associated with HLA-DMB, including cisplatin, dexamethasone, and ethinylestradiol.

**Discussion:**

This study elucidates the function and underlying mechanisms of HLA-DMB in UCEC, providing a potential biomarker and target for immunotherapy in this disease.

## 1 Introduction

Endometrial cancer is the sixth most common cancer among women, with an overall incidence that has risen by 132% over the past 30 years (1). In recent years, uterine corpus endometrial carcinoma (UCEC) has been diagnosed in more than 40,000 women worldwide, with the highest disease burden observed in North America and Western Europe (2). Most patients diagnosed with early-stage endometrial cancer have a high survival rate following treatment, while those diagnosed with late-stage endometrial cancer experience a significantly reduced survival rate (3). In addition, the management of endometrial carcinomas can differ, even if at the same stage, depending on the different guidelines applied in the various geographical areas (4). Transvaginal ultrasound and endometrial biopsy are common methods for diagnosing endometrial cancer, and additional immunohistochemical (IHC) analyses can aid in identifying early uterine corpus endometrial carcinoma (UCEC) (5, 6). However, there is currently no routinely used biomarker for diagnostic or prognostic purposes in UCEC. With the establishment of new genomic classifications of endometrial cancers, the use of biomarkers to guide therapeutic approaches will be a cornerstone of individualized cancer care in the coming decades (7). The molecular algorithm of endometrial cancer plays a pivotal role in prognostication and therapeutic decision-making. By enabling the prediction of recurrence risk and survival rates, it assists clinicians and patients in selecting optimized surgical strategies tailored to the individual patient’s molecular profile. The Betella algorithm exemplifies this approach by categorizing EC into distinct risk categories based on its molecular characteristics, thereby guiding the assignment of specific adjuvant therapies. Such molecular algorithms are indispensable for enhancing the personalized treatment of endometrial cancer, ensuring that therapeutic interventions are both appropriate and effective. This integration of molecular diagnostics into clinical decision-making processes underscores a significant advancement in the management of endometrial cancer (8–10).

The Major Histocompatibility Complex (MHC) is a group of genes that encode major histocompatibility antigens in animals. MHC class II (MHC-II) molecules are expressed on antigen-presenting cells (APCs) and function by binding antigenic peptides and presenting these peptides to antigen-specific CD4+ T cells (11). HLA-DM plays a key role in MHC II antigen presentation and CD4+T cell epitope selection (12). HLA-DMB, the β chain of the MHC class II protein HLA-DM, can regulate the expression of the HTLV-1 protein by modulating the accumulation of autophagosomes, suggesting a novel mechanism for host cells to resist HTLV-1 infection (13). Tumor cell expression of HLA-DMB has been shown to correlate with an increased number of tumor-infiltrating CD8+ T lymphocytes, both of which are associated with improved survival in advanced serous ovarian cancer (14). Additionally, upregulation of the HLA-DMB gene has been linked to survival in cervical cancer patients (15). However, the expression profile, prognostic significance, and molecular mechanisms of HLA-DMB in uterine corpus endometrial carcinoma (UCEC) remain unclear. In this study, we analyze the role of the HLA-DMB gene in endometrial cancer using the database, aiming to identify valuable predictive biomarkers for endometrial cancer and provide direction for its immunotherapy and targeted therapy.

## 2 Materials and methods

### 2.1 Raw data acquisition and processing

Transcriptome RNA-seq data of 589EC cases (554 tumor samples and 35 controls) and corresponding clinical information were obtained from the TCGA database (https://portal.gdc.cancer.gov/). The public HLA-DMB expression data for normal tissue is sourced from the Genotype-Tissue Expression (GTEx) project (GTEx: https://commonfund.nih.gov/GTEx/). The raw data were analyzed using R software (version 4.2.1).

### 2.2 Tumor immunity estimation resource and TSIDB database analysis

TIMER2.0 (http://timer.cistrome.org/) employs six advanced algorithms to provide a more robust estimation of immune infiltration levels within The Cancer Genome Atlas (TCGA) cohort. It can also be employed to investigate gene expression, clinical outcomes, somatic mutations, and somatic copy number alterations (16). TIMER2.0 The tool is structured into three main components: immune association, cancer exploration, and immune estimation, which collectively facilitate the investigation of the relationship between immune infiltration and cancer characteristics in the TCGA dataset. This study utilized the TIMER2.0 platform to analyze the differential expression of HLA-DMB across various cancers and to examine the correlation between gene expression and immune cell infiltration. TISIDB (TISIDB (hku.hk) allows for the exploration of the relationship between target genes and tumor-immune system interactions through high-throughput data analysis, which aids in elucidating the pathogenesis of cancer and developing new immunotherapy strategies (17).

### 2.3 Gene Expression Profiling Interactive Analysis and the Human Protein Atlas databases were utilized for comprehensive analyses

GEPIA2 (http://gepia2.cancer-pku.cn/) primarily utilized for gene expression and survival analyses, with data derived from TCGA and GTEx samples (18). In our study, we examined the expression of HLA-DMB in endometrial cancer (EC) using the expression “DIY module”. Furthermore, using the “survival analysis module”, we explored the association between HLA-DMB expression levels and overall survival (OS) and disease-free survival (DFS) in UCEC. HPA(https://www.proteinatlas.org/) is primarily used to assess protein expression in endometrial cancer and normal tissues.

### 2.4 UALCAN analysis

UALCAN (http://ualcan.path.uab.edu) provides access to pre-computed gene and protein expression data, promoter DNA methylation status, and Kaplan-Meier survival analyses based on tumor subgroups. It offers intuitive visualization capabilities (19). In this study, UALCAN was utilized to analyze the transcript levels of HLA-DMB across different types of endometrial cancer.

### 2.5 Survival analysis

RNA sequencing data (FPKM) from the TCGA database and the TCGA-UCEC project were downloaded and processed, with normal and non-clinical samples removed. The survival package was utilized to perform proportional hazards hypothesis testing and fit survival regression, and the results were visualized using the survminer and ggplot2 packages (20). All data analyses were conducted using R software (version 4.2.1).

### 2.6 Protein interactions network and module analysis

The STRING database is utilized to integrate all known and predicted associations between proteins, including both physical interactions and functional associations (21). Protein-protein interaction (PPI) analysis plays a crucial role in the fields of transcriptomics and proteomics. In this investigation, we utilized PPI analysis to elucidate the interactions among proteins, which are depicted in the form of network diagrams. This approach aids in understanding the complex interplay and functional coordination within the cellular environment. In this study, it was primarily used to predict proteins closely related to HLA-DMB expression.

### 2.7 KEGG pathway enrichment analysis and GSEA for HLA-DMB

The Gene Ontology (GO) database classifies gene functions into three categories: biological process (BP), cellular component (CC), and molecular function (MF) (22). The KEGG database (https://www.kegg.jp) is organized into systematic, genomic, chemical, and health information categories, containing various biological objects. Functional linkages between genes can be established through relevant pathway modules (23). The Gene Set Enrichment Analysis (GSEA) algorithm is employed to identify categories of gene and protein overexpression that are associated with disease phenotypes (24). In this study, the KEGG database and the GSEA algorithm were used to obtain a list of genes closely related to HLA-DMB expression. The predefined gene set was sourced from the Molecular Signature Database (MSigDB) (https://www.gsea-msigdb.org/gsea/msigdb/index.jsp). MSigDB is a comprehensive repository of well-annotated gene collections that are commonly utilized to identify key genes and pathways involved in various biological processes. In this study, we explored the enriched pathways associated with HLA-DMB in endometrial cancer using the annotated gene sets from the MSigDB. A *p*-value of <0.05 was considered significant for enrichment analysis.

### 2.8 The CTD database was used to explore the chemical drugs related to HLA-DMB

The Comparative Toxicogenomics Database (CTD; http://ctdbase.org/) Links genes, phenotypes, diseases, and chemicals, enabling the exploration of chemicals related to target genes and the construction of potential chemical-disease mechanism pathways (25).We mainly used the CTD database to analyze the chemicals associated with HLA-DMB in endometrial cancer.

### 2.9 collection of clinical samples

Endometrial cancer samples and adjacent normal tissues were collected from twenty-three patients who underwent hysterectomy at the Second Hospital of Hebei Medical University. All specimens were stored in tissue-specimen fixative, and the cells were subsequently isolated and cultured. None of these patients received preoperative chemotherapy or radiotherapy and had no other comorbidities aside from endometrial cancer. The study was approved by the hospital ethics committee, and all patients provided informed consent.

### 2.10 Reverse transcription-quantitative polymerase chain reaction

Total RNA was extracted using Trizol, and complementary DNA (cDNA) was synthesized with a reverse transcription kit (Wuhan Sevier Biotechnology Co., Ltd., China), followed by quantitative reverse transcription polymerase chain reaction (qRT-PCR) using the SYBR Green PCR kit (Wuhan Sevier Biotechnology Co., Ltd., China) and a fluorescent quantitative PCR apparatus (Bio-Rad, USA). This study was performed in triplicate, with GAPDH used as an internal control. The real-time PCR reaction system consisted of 7.5 μL of 2× Universal Blue SYBR Green qPCR Master Mix, 1.5 μL of 2.5 μM gene primers (upstream and downstream), 2.0 μL of reverse transcription product (cDNA), and 4.0 μL of nuclease-free water. The reaction conditions included predenaturation at 95°C for 30 seconds, followed by 40 cycles of denaturation at 95°C for 15 seconds and annealing/extension at 60°C for 30 seconds. The forward primer sequence for HLA-DMB was 5’-GACATACCAGACCCTCTCCCATTTA-3’, and the reverse primer sequence was 5’-TTGGACCCAGGAAGAGGAGTGT-3’. The forward primer sequence for GAPDH was 5’-GGAAGCTTGTCATCAATGGAAATC-3’, and the reverse primer sequence was 5’-TGATGACCCTTTTGGCTCCC-3’. Relative gene expression was calculated using the 2−ΔΔCT method.

### 2.11 Immunohistochemical verification of the protein expression of HLA-DMB

Clinical tissue wax blocks were sectioned, separated, hydrated, and subjected to antigen retrieval in a 98°C antigen repair solution for 5 minutes before cooling to room temperature. Each paraffin section was incubated with 50 μL of endogenous peroxidase blocker (Wuhan Sevier Biotechnology Co., Ltd., China) for 25 minutes and rinsed with phosphate-buffered saline (PBS) at pH 7.4. The primary antibody was blocked with rabbit serum for 30 minutes, while other antibodies were blocked with 3% bovine serum albumin (BSA) for 30 minutes. After removing the blocking solution, the primary antibody, prepared in PBS at a dilution of 1:800, was added dropwise, and the cells were incubated in a wet box at 4°C overnight. After washing three times, an HRP-labeled secondary antibody was added, and the cells were incubated at room temperature for 50 minutes. Following DAB (G1212, Wuhan Sevier Biotechnology Co., Ltd., China) development and hematoxylin staining, photographs were taken under a microscope.

### 2.12 Statistical analysis

Bioinformatics statistical analyses were conducted using public bioinformatics databases and R software (version 4.2.1). GraphPad Prism 9.0 was employed for PCR analysis. Student’s t-test or non-parametric tests were used for comparisons between groups. The Pearson χ² test was applied to analyze the relationship between HLA-DMB expression and clinicopathological variables. In survival analysis, the Kaplan-Meier (K-M) method estimates survival probability, the log-rank test compares two or more survival curves, and the Cox proportional hazards regression model analyzes the influencing factors on outcomes. Cox proportional hazards regression models investigate the association between patient characteristics and survival time based on one or more predictor variables, while also assessing the impact of multiple factors on survival simultaneously. The survival prediction ability of HLA-DMB was evaluated using the receiver operating characteristic (ROC) curve and area under the curve (AUC). The hazard ratio (HR) with a 95% confidence interval (CI) was calculated to estimate the hazard risk of individual factors. R was used to create the nomogram and build the prediction model. A *p*-value of < 0.05 indicates statistical significance, while a *p*-value of < 0.01 indicates high statistical significance. All reported *p*-values were two-sided (26).

## 3 Results

### 3.1 Analysis of HLA-DMB mRNA and protein expression

**Figure 1:**
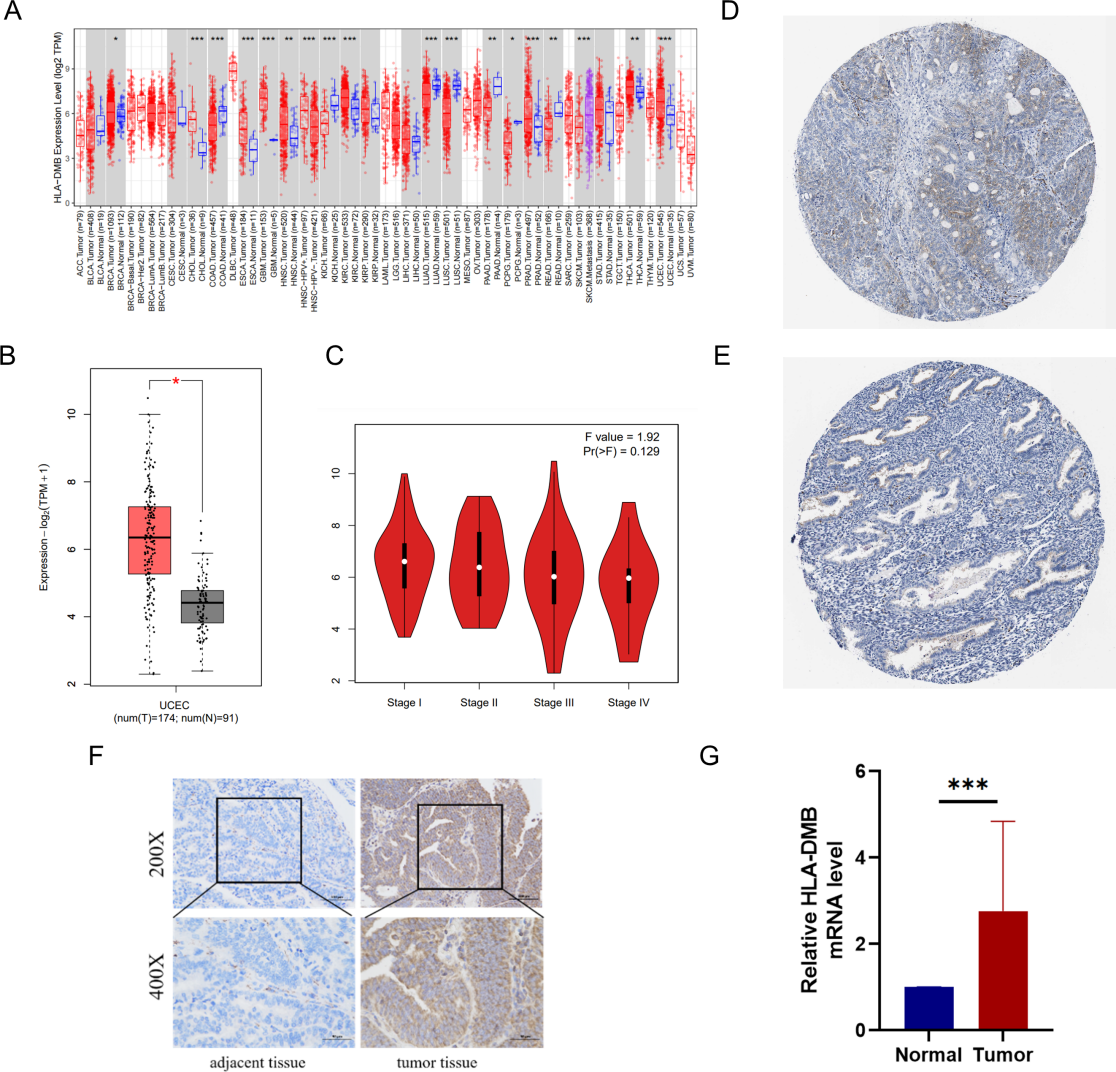
mRNA and protein expression of HLA-DMB in tumor tissues. **(A)** mRNA expression of HLA-DMB in pan-cancer in TIMER2.0. **(B)** mRNA expression of HLA-DMB in EC; and **(C)** correction with clinical stage in GEPIA2. **(D)** protein expression of HLA-DMB in EC; and **(E)** normal tissue in HPA. EC, endometrial cancer; HPA, The Human Protein Atlas. **(F)** Immunohistochemical analysis of HLA-DMB in endometrial cancer tissues and adjacent tissues. **(G)** Expression level of HLA-DMB mRNA in endometrial cancer tissues and adjacent tissues. * p<0.05, ** p<0.01, *** p<0.001.

We utilized several databases to analyze the expression levels of HLA-DMB in tumors. First, the expression level of HLA-DMB in pan-cancer was analyzed using the TIMER2.0 database. The results indicated that HLA-DMB was differentially expressed across various cancers, particularly in endometrial cancer (**Figure 1A**). The GEPIA2 database was employed to confirm the higher expression of HLA-DMB mRNA in UCEC compared to normal tissues, and the relationship between HLA-DMB expression and the stage of endometrial cancer was analyzed (**Figures 1B, C**). Immunohistochemical results for HLA-DMB in endometrial cancer patients and normal human endometrial tissues were obtained from the HPA database, revealing that HLA-DMB protein levels were elevated in endometrial cancer (**Figures 1D, E**). To validate the expression of HLA-DMB, quantitative PCR (qPCR) and immunohistochemistry were performed on four cases of endometrial carcinoma and adjacent normal tissues. The results demonstrated that HLA-DMB mRNA expression was significantly higher in UCEC than in adjacent normal tissues (*p* = 0.0002). In immunohistochemistry, the expression of HLA-DMB protein was also greater than that in adjacent tissues (**Figures 1F, G**).

### 3.2 High expression of HLA-DMB indicates better survival

**Figure 2:**
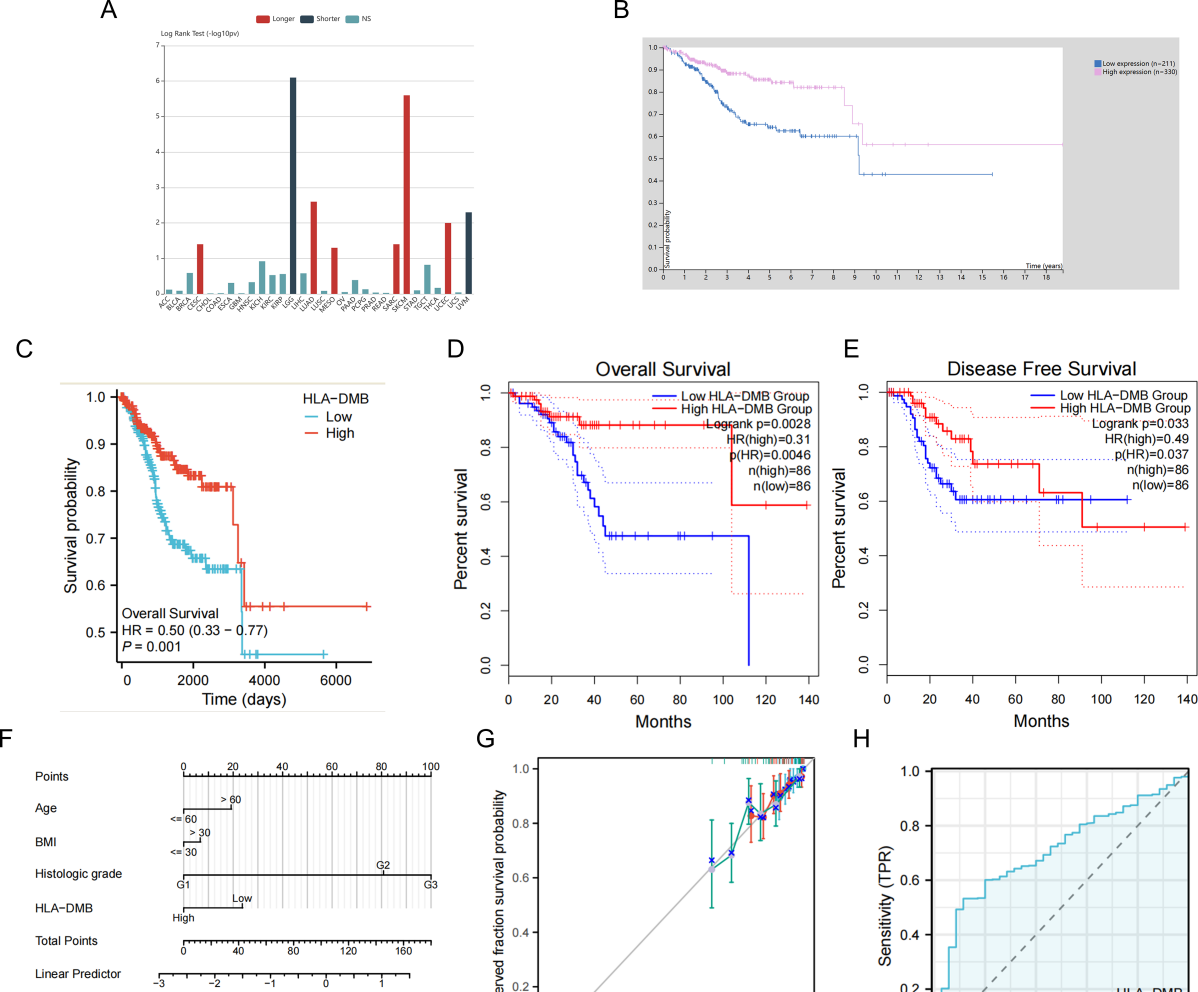
Relationship between HLA-DMB expression and prognosis of UCEC. **(A)** the relationship between HLA-DMB expression and clinical outcome of cancers. **(B)** Kaplan–Meier analysis of overall survival in HPA. **(C)** Kaplan–Meier analysis of overall survival from TCGA. **(D, E)** Overall Survival (OS) and Disease Free Survival (DFS) for patients in GEPIA2. **(F)** A nomogram for predicting the probability of 1-, 2- and 3-year OS in endometrial carcinoma patients. **(G)** Calibration plots validating the efficiency of nomograms for OS. **(H)** ROC curves for predicting the diagnostic power of HLA-DMB.

Given the high expression of HLA-DMB in UCEC compared to normal tissues, we investigated the prognostic value of HLA-DMB in UCEC. We analyzed the relationship between HLA-DMB expression and clinical outcomes. Notably, high expression of HLA-DMB predicted better clinical outcomes in endometrial cancer (**Figure 2A**). Patients were divided into two groups based on the median HLA-DMB expression level. The results indicated that patients with high HLA-DMB expression had a better prognosis than those with low HLA-DMB expression (**Figures 2B–E**). Specifically, higher HLA-DMB expression was associated with better overall survival (OS)(*p*=0.0028), and similarly, higher HLA-DMB expression was associated with better disease-free survival (DFS) (*p*=0.033). It suggests that HLA-DMB may serve as a promising biomarker for predicting survival in endometrial cancer. Subsequently, a nomogram was plotted based on age, body mass index (BMI), histological grade, and HLA-DMB expression level (**Figure 2F**). The calibration curve demonstrated a desirable prediction accuracy of the nomogram for 1-, 2-, and 3-year clinical outcomes (**Figure 2G**). Univariate (HR = 0.503, *p* = 0.001) and multivariate (HR = 0.554, *p* = 0.001) analyses showed that HLA-DMB has independent prognostic value (**Table 1**). We plotted receiver operating characteristic (ROC) curves to assess the diagnostic power of HLA-DMB. The area under the curve (AUC) value for HLA-DMB was 0.711 (CI = 0.600-0.783), indicating that it has certain diagnostic efficacy (**Figure 2H**).

**Table 1:** Univariate and multivariate regression analysis of endometrial cancer.

Characteristics	Total (N)	Univariate analysis		Multivariate analysis	
		Hazard ratio (95% CI)	*P* value	Hazard ratio (95% CI)	*P* value
Clinical stage	553		**< 0.001**		
Stage I&Stage II	394	Reference		Reference	
Stage III&Stage IV	159	3.553 (2.362 - 5.344)	**< 0.001**	2.874 (1.845 - 4.476)	**< 0.001**
Histological type	553		**< 0.001**		
Endometrioid	411	Reference		Reference	
Mixed	24	2.428 (1.040 - 5.672)	**0.040**	1.421 (0.594 - 3.397)	0.430
Serous	118	2.674 (1.744 - 4.100)	**< 0.001**	1.209 (0.736 - 1.986)	0.454
Histologic grade	542		**< 0.001**		
G1&G2	220	Reference		Reference	
G3	322	3.298 (1.917 - 5.672)	**< 0.001**	2.165 (1.194 - 3.926)	**0.011**
HLA-DMB	553		**0.001**		
Low	276	Reference		Reference	
High	277	0.503 (0.329 - 0.769)	**0.001**	0.554 (0.353 - 0.868)	**0.010**

The bold value in the table indicates that the data are statistically significant (P < 0.05).

### 3.3 Correlation between HLA-DMB expression and clinical features

**Figure 3:**
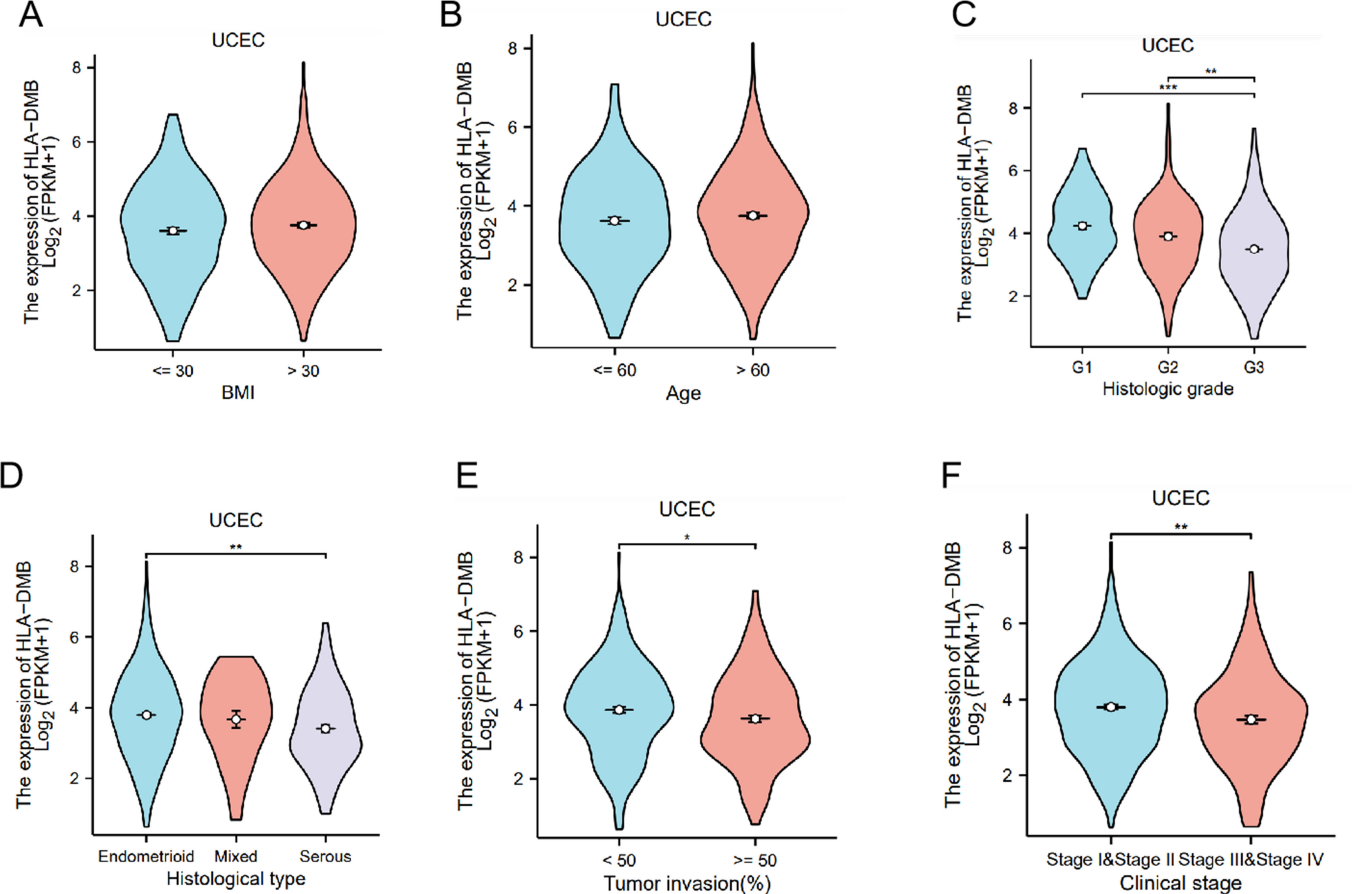
Correlation between HLA-DMB expression and clinical features. **(A)** Comparison of HLA-DMB expression in patients with BMI ≤ 30 and > 30; **(B)** Comparison of HLA-DMB expression in patients with age ≤ 60 and >60; **(C)** Comparison of HLA-DMB expression in patients with different histological types; **(D)** Comparison of HLA-DMB expression with different histological grades; **(F)** Comparison of HLA-DMB expression in patients with tumor invasion ≤ 50% and >50%; **(G)** Comparison of HLA-DMB expression in patients with clinical stages. * p<0.05, ** p<0.01, *** p<0.001.

Additionally, we analyzed the relationship between HLA-DMB expression and clinical features, including body mass index (BMI), age, histological type, histological grade, tumor invasion, and clinical stage. The results indicated that HLA-DMB expression was significantly correlated with tumor histological type, histological grade, tumor invasion, and clinical stage (**Figure 3**). Logistic regression analysis revealed that HLA-DMB expression was lower in stages III and IV compared to stages I and II (*P* = 0.019), lower in grade 3 (G3) than in grades 1 (G1) and 2 (G2) (*P* < 0.001), and lower in tumors with invasion depth ≥50% compared to those with invasion depth <50% (**Table 2**).

**Table 2:** Association between HLA-DMB expression and clinicopathologic parameters by logistic regression.

Characteristics	Total (N)	OR(95%CI)	*P* value
Clinical stage (Stage III & Stage IV vs. Stage I & Stage II)	554	0.642(0.442-0.930)	**0.019**
Age (>60 vs. <=60)	551	1.179(0.835-1.665)	0.350
Histologic grade (G3 vs. G1&G2)	543	0.449(0.316-0.691)	**<0.001**
Tumor invasion(%) (>=50 vs. <50)	476	0.478(0.331-0.691)	**<0.001**
Diabetes (Yes vs. No)	453	1.064(0.704-1.610)	0.768
BMI (>30 vs. <=30)	521	0.981(0.691-1.391)	0.913
Menopause status (Post vs. Pre&Peri)	507	1.174(0.661-2.085)	0.584

The bold value in the table indicates that the data are statistically significant (P < 0.05).

### 3.4 HLA-DMB expression is related to the immune microenvironment of UCEC

**Figure 4:**
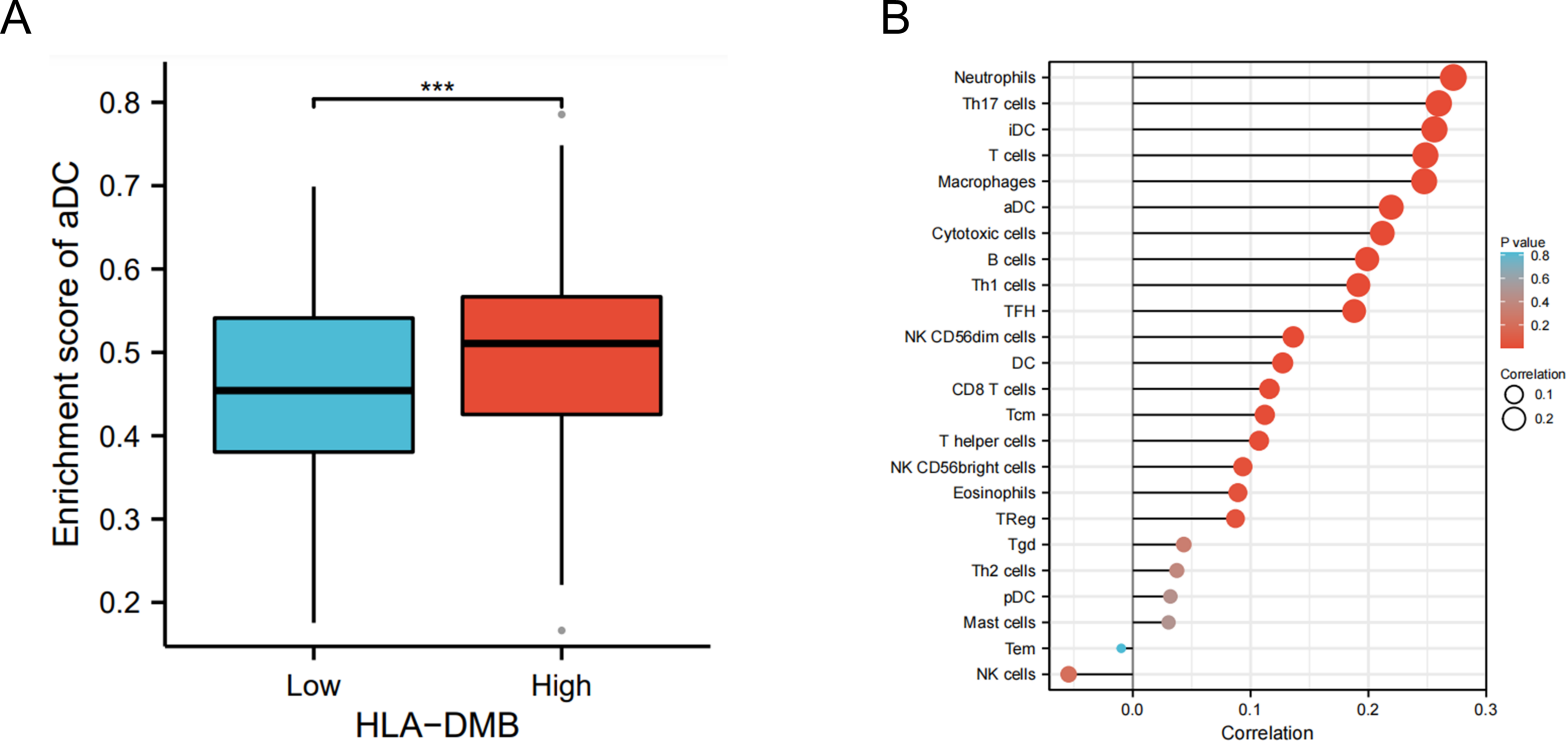
HLA-DMB expression is related to the immune infiltration of UCEC. **(A)** Relationship between HLA-DMB expression levels and immune infiltration scores; **(B)** Correlation between HLA-DMB expression and immune cells. *** p<0.001.

**Figure 5:**
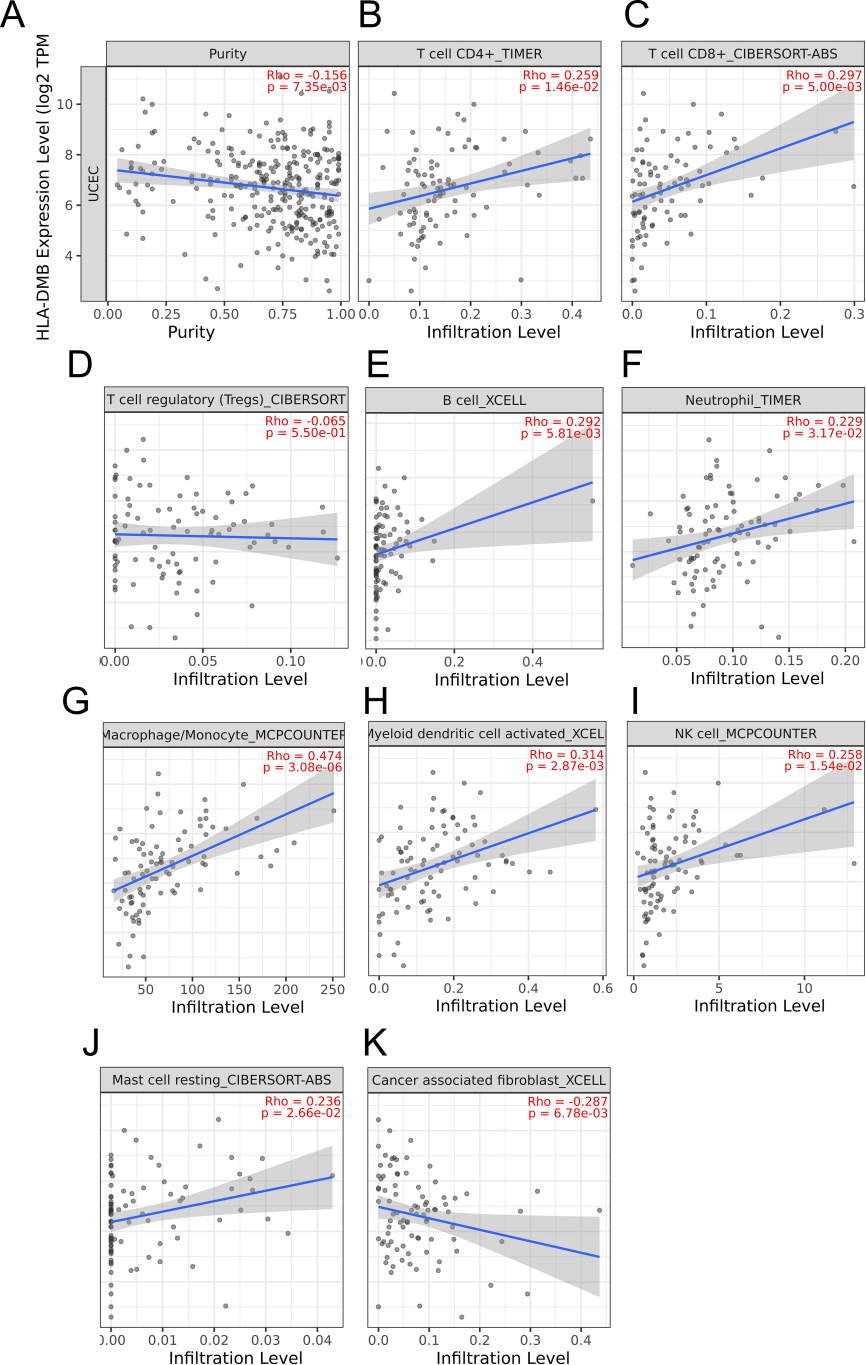
The relationship between HLA-DMB expression and tumor purity **(A)**, infiltration of T cell CD4+ **(B)**, T cell CD8+ **(C)**,T cell regulatory **(D)**,B cell **(E)**, neutrophil **(F)**, macrophage/monocyte **(G)**, myeloid dendritic cell activated **(H)**, NK cell **(I)**, Mast cell resting **(J)** and cancer associated fibroblast **(K)** in TIMER2.0 database.

**Figure 6:**
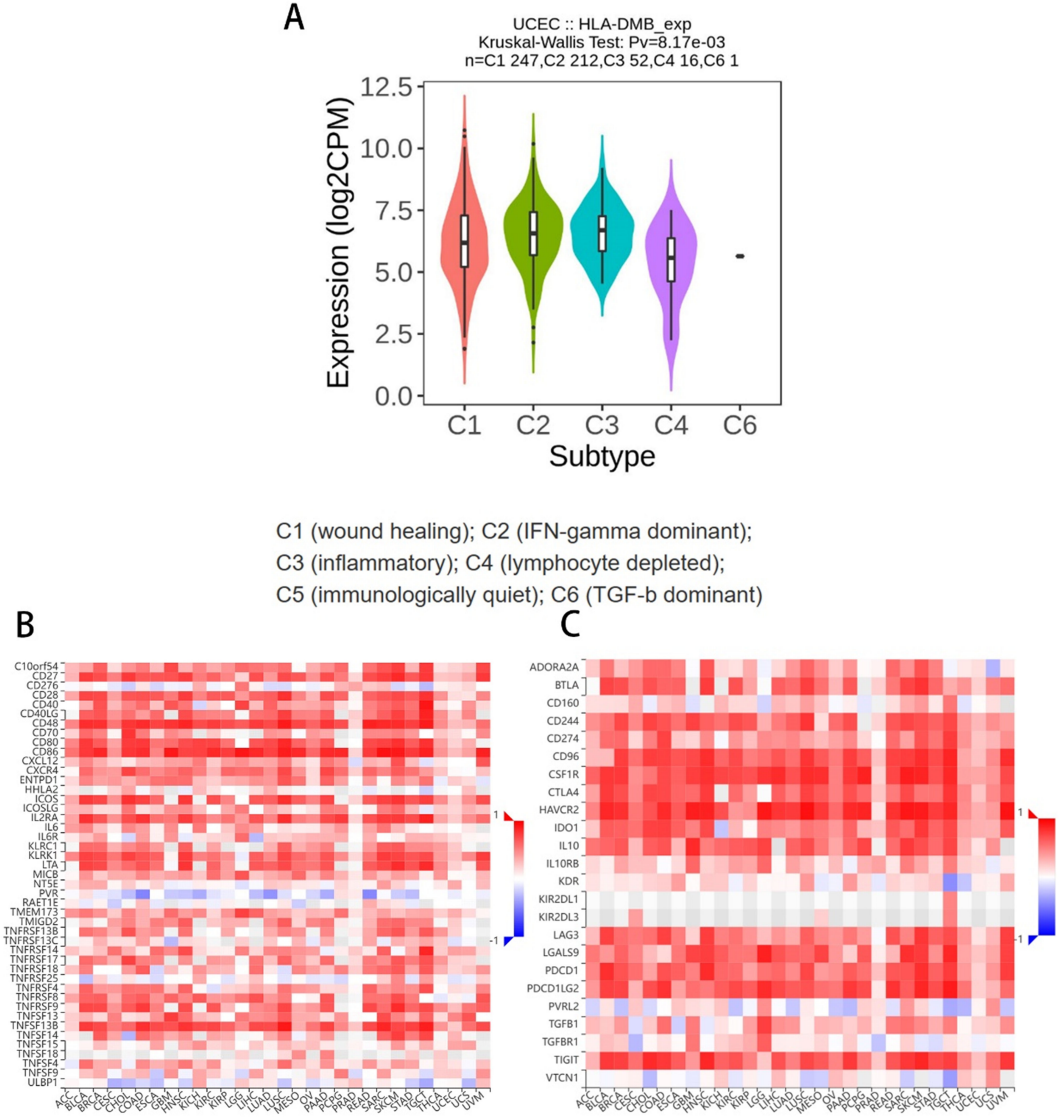
Relationship between HLA-DMB expression and the immune microenvironment **(A)** Molecular subtypes associated with HLA-DMB expression in endometrial cancer; **(B)** Relationship between HLA-DMB and immune enhancing factors; **(C)** Relationship between HLA-DMB and immunosuppressive factors.

Based on the above results, we aimed to investigate the correlation between HLA-DMB expression and the immune microenvironment of UCEC using the single-sample Gene Set Enrichment Analysis (ssGSEA) algorithm. This algorithm was employed to estimate the relationship between HLA-DMB expression and 24 immune cell types (**Figure 4A**). Samples with high HLA-DMB expression exhibited higher enrichment scores for immune cells. The results indicated that HLA-DMB expression was most positively correlated with neutrophils, Th17 cells, immature dendritic cells (iDC), and T cells, while it was negatively correlated with effector memory T (Tem) cells and natural killer (NK) cells (**Figure 4B**). We further validated the correlation between HLA-DMB expression and various immune cells using the TIMER2.0 database (**Figures 5A–K**). HLA-DMB expression was negatively correlated with tumor purity and positively correlated with the infiltration levels of CD4+ T cells, CD8+ T cells, B cells, neutrophils, monocytes, myeloid dendritic cells, NK cells, and mast cells. However, it was negatively correlated with regulatory T cells (Tregs) and cancer-associated fibroblasts. In endometrial cancer, HLA-DMB expression was primarily associated with wound healing, interferon-gamma dominant, inflammatory, and lymphocyte-depleted molecular subtypes, but less so with immunologically quiet and transforming growth factor-beta dominant subtypes (**Figure 6A**). Further analysis of the relationship between HLA-DMB and immune-enhancing factors, as well as immunosuppressive factors, revealed that HLA-DMB was positively correlated with several immune-enhancing factors, such as NT5E, TMEM173, TNFRSF14, and TNFSF13 in UCEC (**Figure 6B**), but not correlated with various immunosuppressive factors (**Figure 6C**). These results suggest that HLA-DMB may be associated with anti-tumor immunity in endometrial cancer.

### 3.5 Analysis of genetic alterations of HLA-DMB in UCEC

**Figure 7:**
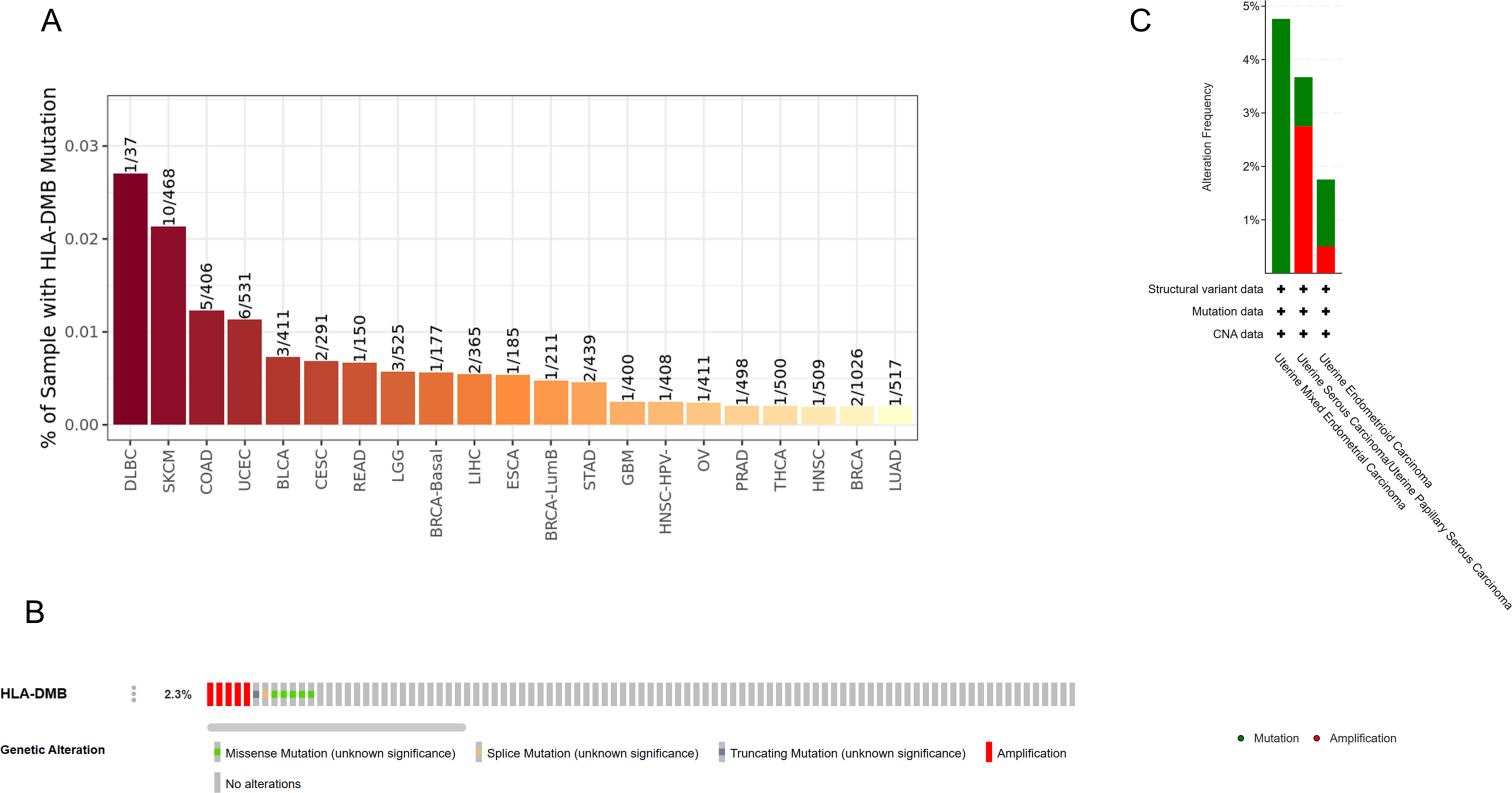
Analysis of genetic alterations of HLA-DMB **(A)** Mutational profile of HLA-DMB in pan-cancer; **(B, C)** Mutation rate of HLA-DMB in UCEC.

We further analyzed the prevalence of HLA-DMB mutations in various cancers and found that the tumors with the highest HLA-DMB mutation rates were diffuse large B-cell lymphoma (DLBC), melanoma (SKCM), colon cancer (COAD), and UCEC (**Figure 7A**). The mutation rate of HLA-DMB in UCEC was 2.3%, with the major mutation types being amplification, truncating mutations, splice mutations, and missense mutations (**Figure 7B**). Additionally, we found that uterine mixed endometrioid carcinoma was most prone to HLA-DMB mutations, followed by endometrial serous carcinoma, which primarily exhibited amplification mutations, and finally endometrioid carcinoma (**Figure 7C**).

### 3.6 Analysis of HLA-DMB co-expression, Gene Ontology and Gene Set Enrichment Analysis

**Figure 8:**
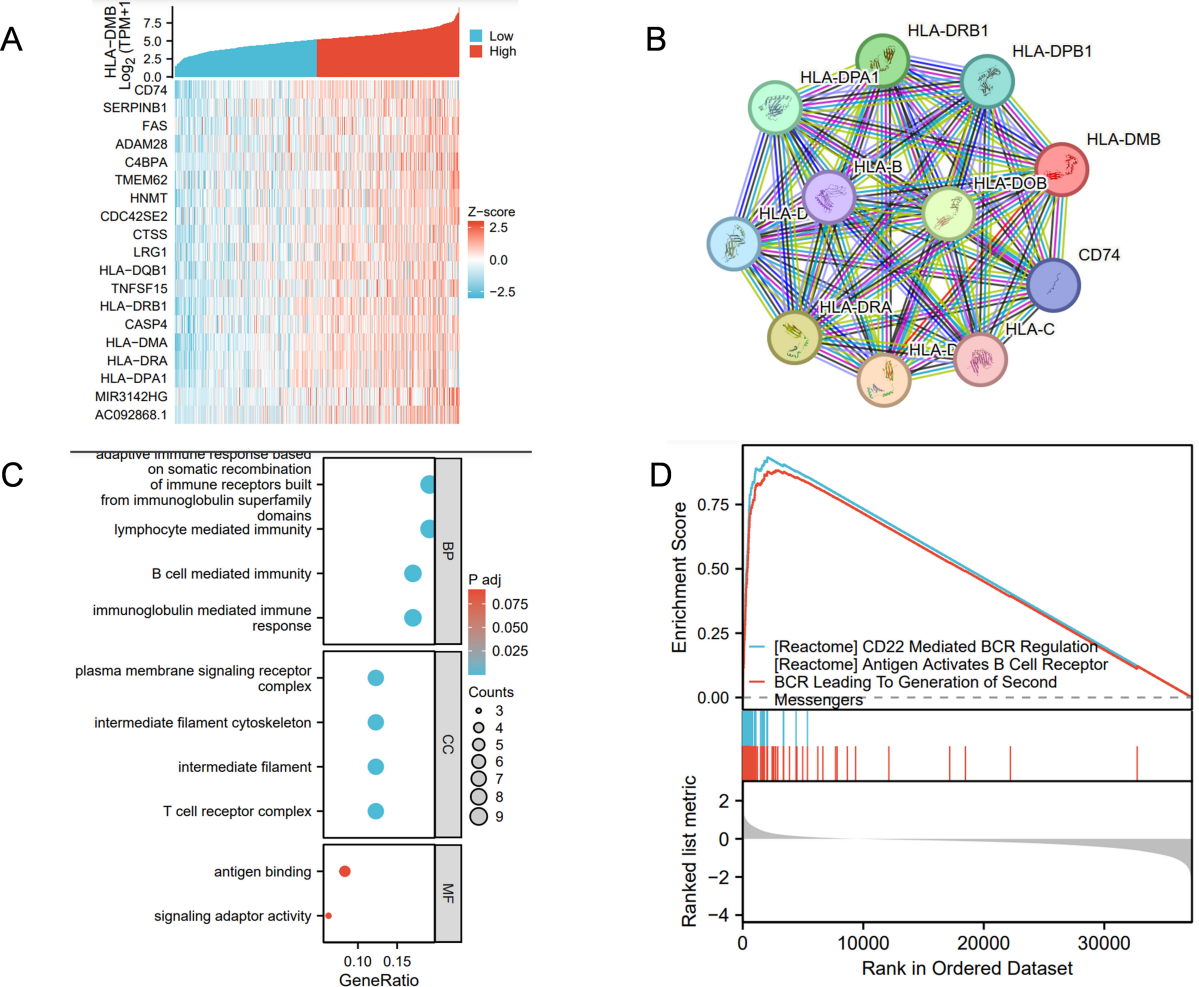
Analysis of HLA-DMB Co-expression, Gene Ontology (GO) and Gene set enrichment analysis (GSEA) **(A)** The genes associated with HLA-DMB expression; **(B)** PPI interaction network analysis; **(C)** Analysis of HLA-DMB Gene Ontology (GO); **(D)** GSEA pathway analysis of HLA-DMB in endometrial cancer.

Co-expression analysis of HLA-DMB revealed that CD74, SERPINB1, FAS, ADAM28, and C4BPA were most closely associated with HLA-DMB, as shown in the heat map (**Figure 8A**). The STRING database was utilized to analyze the top 10 proteins that interact with HLA-DMB, which included CD74, HLA-DOB, HLA-DPB1, HLA-DRB1, HLA-DPA1, HLA-B, HLA-DRA, HLA-DMA, HLA-C, and HLA-D (**Figure 8B**). We then conducted Gene Ontology (GO) analysis, which indicated that the genes were primarily enriched in adaptive immune response based on somatic recombination of immune receptors derived from immunoglobulin superfamily domains, lymphocyte-mediated immunity, B cell-mediated immunity, and immunoglobulin-mediated immune response during the biological process (BP). Regarding the cellular component (CC), these genes were primarily associated with the plasma membrane signaling receptor complex, intermediate filament cytoskeleton, intermediate filaments, and T cell receptor complex. In terms of molecular function (MF), these genes were mainly involved in antigen binding and signaling adaptor activity (**Figure 8C**). Based on these results, we conducted Gene Set Enrichment Analysis (GSEA) pathway analysis and found that HLA-DMB is primarily involved in CD22-mediated B cell receptor (BCR) regulation, which leads to antigen activation of the BCR and the generation of second messengers (**Figure 8D**).

### 3.7 Chemicals associated with HLA-DMB gene in EC estimated by the CTD database

To identify chemicals related to HLA-DMB and provide insights for the treatment of endometrial cancer, we retrieved data from the Comparative Toxicogenomics Database (CTD) and found three substances associated with HLA-DMB: cisplatin, dexamethasone, and ethinyl estradiol. Among these, the combination of cisplatin and Jinfukang increased HLA-DMB mRNA expression, while the combination of cisplatin and panobinostat may also influence HLA-DMB expression. However, dexamethasone resulted in decreased expression of HLA-DMB mRNA, whereas ethinyl estradiol led to increased expression of HLA-DMB mRNA (**Table 3**).

**Table 3:** Literature queries on drugs interacting with HLA-DMB based on CTD database.

Chemical Name	Chemical ID	Interaction	Reference Count
Cisplatin	D002945	[Cisplatin co-treated with jinfukang] results in increased expression of HLA-DMB mRNA	(27)
		[Cisplatin co-treated with Panobinostat] affects the expression of HLA-DMB mRNA	(28)
Dexamethasone	D003907	Dexamethasone results in decreased expression of HLA-DMB mRNA	(29)
Ethinyl Estradiol	D004997	Ethinyl Estradiol results in increased expression of HLA-DMB mRNA	(30)

## 4 Conclusion

With the aging population and the rising prevalence of obesity, the incidence of endometrial cancer is increasing annually, making it the most common gynecological cancer in developed countries (1). The survival rates for patients with recurrent and advanced endometrial cancer have not improved significantly in recent decades. Molecular biology research aims not only to identify predictive biomarkers but also to provide directions for targeted therapy and immunotherapy for endometrial cancer, ultimately facilitating individualized treatment for patients (1, 31, 32).

CLIP proteins, or class II-associated invariant chain peptides, are critical components in the immune system, playing a key role in antigen presentation and the immune response. Specifically, CLIP peptides are found on MHC class II molecules. When MHC class II molecules bind to antigens, they associate with a molecule called the invariant chain, forming a complex. Within this complex, CLIP peptides are part of the invariant chain and function to block the binding of other antigens to the MHC class II molecules. This mechanism helps regulate and protect MHC class II molecules from premature antigen binding. Radiochemotherapy (RCT) can induce cervical cancer with innate immune activation and MHC class II expression, revealing the complex interactions within the tumor ecosystem during RCT (33). Additionally, MHC class II molecules involved in the immune response have been found to exert a wide variety of tumor and anti-tumor effects (33–35). In the present study, HLA-DMB, as part of the MHC class II complex presenting antigens, demonstrates significant tumor-suppressive effects in endometrial cancer. Increasing evidence suggests that the upregulation of HLA-DMB is associated with higher survival rates in advanced serous ovarian cancer and cervical cancer, although the mechanisms of action differ. However, the role of HLA-DMB in endometrial carcinoma and the underlying mechanisms remain unclear, and its function in the tumor immune microenvironment has yet to be explored.

In this study, we utilized TCGA and other databases to analyze the differential expression of HLA-DMB across 33 types of cancer and adjacent tissues. Tumor tissues exhibited higher expression levels than normal tissues in BRCA, CHOL, ESCA, GBM, HNSC, KIRC, KIRP, PRAD, THCA, and UCEC. Notably, the expression of HLA-DMB was higher in HPV-positive HNSC compared to HPV-negative HNSC. To further investigate the role of HLA-DMB in endometrial cancer, we verified the high expression of the HLA-DMB gene and protein in the endometrium. We then explored the relationship between HLA-DMB expression and cancer prognosis, finding that high expression levels in CESC (cervical squamous cell carcinoma), LUAD (lung adenocarcinoma), MESO (mesothelioma), SERC (renal cell carcinoma), SKCM (renal cell carcinoma), and UCEC were associated with better clinical outcomes. Further analysis revealed that the high expression group of HLA-DMB in endometrial cancer had improved overall survival (OS) and disease-free survival (DFS). Our data suggest that HLA-DMB is significantly downregulated in UCEC patients with poor clinical outcomes. In the subgroup analysis, HLA-DMB was significantly down-regulated in patients with higher histological grade (especially G3), serous cancer type, higher depth of tumor invasion (≥50%), and advanced clinical stage (stage III and IV). We analyzed the role of HLA-DMB in the immune microenvironment of endometrial cancer and found that the high expression group exhibited higher immune infiltration scores and was positively correlated with various immune cells, particularly monocytes. Additionally, we discovered that HLA-DMB was positively correlated with immune-enhancing factors, with TNFSF13 being the most closely related factor. These data suggest that HLA-DMB plays a significant role in the immune response in endometrial cancer and may be associated with prolonged patient survival. The molecular characteristics of UCEC were further investigated based on the differential expression levels of HLA-DMB. It was found that HLA-DMB is frequently genetically altered in endometrial carcinoma, particularly in mixed endometrioid carcinoma. Expression analysis revealed a high correlation between the expression of HLA-DMB and five genes: CD74, SERPINB1, FAS, ADAM28, and C4BPA. Among these, CD74 exhibited the highest correlation with HLA-DMB. Regulatory changes in CD74 and HLA-DM have been shown to lead to significant remodeling of the tumor microenvironment. Furthermore, the involvement of CD74 can enhance the cytotoxic effects of traditional chemotherapy drugs on anaplastic large cell lymphoma (ALCL) cell lines (36, 37). Fas ligand (FasL) is a molecule that can induce apoptosis in Fas-positive target cells. Tumor cells may evade destruction by the immune system through the expression of Fas ligand (38, 39). GO analysis indicated that HLA-DMB is involved in lymphocyte-mediated immunity, B cell-mediated immunity, and immunoglobulin-mediated immune responses. GSEA pathway analysis revealed that HLA-DMB primarily participates in CD22-mediated B cell receptor (BCR) regulation, which leads to the modulation of second messengers and complement cascades. This process plays a crucial role in mediating inflammation and immunity (40–43). Based on the above analysis, it is reasonable to speculate that HLA-DMB may play a role in inhibiting endometrial cancer by participating in the immune response and altering the tumor immune microenvironment. In our pharmaceutical analysis, we found that the chemical drugs associated with HLA-DMB include cisplatin, dexamethasone, and ethinylestradiol. Additionally, cisplatin combined with JinFukang can regulate gene-related signaling pathways involved in apoptosis. Dexamethasone can enhance the expression of HLA-DMB, while ethinylestradiol can further increase HLA-DMB expression. This evidence supports the potential for targeted therapy involving HLA-DMB and immunotherapy (30, 44, 45). In this study, we evaluated the cost-effectiveness of HLA-DMB testing. First, direct costs include the expenses associated with testing reagents and equipment, as well as the labor costs for laboratory operations. Indirect costs encompass patient time and transportation expenses. By analyzing the potential value of HLA-DMB in the diagnosis and treatment of endometrial cancer, we found that it can improve patient survival rates, effectively reducing overall medical costs and providing long-term economic benefits. In conclusion, HLA-DMB testing demonstrates significant potential value in the clinical diagnosis and treatment of endometrial cancer, as evidenced by its favorable cost-benefit ratio.

This study, while providing valuable insights, does possess certain limitations that warrant further exploration. Most notably, although our analysis has convincingly shown that high expression levels of HLA-DMB are prevalent in endometrial cancer and are predictive of favorable survival outcomes for patients, the precise mechanisms by which HLA-DMB influences endometrial cancer remain inadequately understood. Specifically, further research is imperative to delineate the effects of HLA-DMB on tumor behavior and its role in modulating the immune response. Comprehensive elucidation of these aspects will contribute significantly to our understanding of the potential therapeutic applications and immune-related dynamics of HLA-DMB in the context of endometrial cancer.

In conclusion, our study demonstrated the prognostic value of HLA-DMB in UCEC and provided insights into the significant role of the immune microenvironment associated with HLA-DMB. Additionally, we thoroughly discussed the expression of genes related to HLA-DMB. We identified HLA-DMB as a promising biomarker for endometrial cancer patients and its involvement in anti-tumor immune responses. Furthermore, through drug sensitivity analysis, we found that the drugs closely related to the HLA-DMB gene include cisplatin, dexamethasone, and ethinylestradiol, offering a new perspective for the treatment of endometrial cancer.

## Data Availability

Publicly available datasets were analyzed in this study. All publicly available datasets in this work are available from TCGA (https://portal.gdc.cancer.gov/) and Genotype-Tissue Expression (GTEx)project (GTEx: https://commonfund.nih.gov/GTEx/k).T; further inquiries can be directed to the corresponding author.
